# Significant advantages for first line treatment with TNF-alpha inhibitors in pediatric patients with inflammatory bowel disease – Data from the multicenter CEDATA-GPGE registry study

**DOI:** 10.3389/fped.2022.903677

**Published:** 2022-07-19

**Authors:** Merle Claßen, Jan de Laffolie, Martin Claßen, Alexander Schnell, Keywan Sohrabi, André Hoerning

**Affiliations:** ^1^Clinic for Children and Adolescent Medicine, Friedrich Alexander University Erlangen-Nuremberg, Erlangen, Germany; ^2^Abteilung für Allgemeine Pädiatrie und Neonatologie, Justus Liebig University Gießen, Gießen, Germany; ^3^Klinik für Kinder- und Jugendmedizin, Klinikum Bremen-Mitte, Bremen, Germany

**Keywords:** Crohn’s disease, ulcerative colitis, anti-TNF-α inhibitor therapy, first-line therapy, pediatric inflammatory bowel disease (IBD)

## Abstract

**Background and aims:**

In recent years, biological agents, such as anti-TNF-α blockers, have been introduced and have shown efficacy in pediatric patients with inflammatory bowel disease (IBD). Here, the prescription mode differentiated into a first/second line application, and efficacy and side effects are evaluated beginning from 2004 until today.

**Methods:**

Statistical analyses of the prospective and ongoing CEDATA multicenter registry data from the Society of Pediatric Gastroenterology and Nutrition (GPGE) were performed for patients receiving a biological agent at least once during the period from June 2004 until November 2020 (*n* = 487). The analyzed parameters were patient demographics, disease extent and behavior, prior or concurrent therapies, duration and outcome of biological therapy, disease-associated complications, drug-related complications, laboratory parameters and treatment response as determined by the Physician’s Global Assessment.

**Results:**

Crohn’s disease (CD) was present in 71.5% of patients, and 52% were boys. Patients showed high disease activity when receiving a first-line TNF-α blocker. After 2016, patients who failed to respond to anti-TNF-α induction therapy were treated with off-label biologics (vedolizumab 4.3% and ustekinumab 2.1%). Propensity score matching indicated that patients with CD and higher disease activity benefitted significantly more from early anti-TNF-α therapy. This assessment was based on a clinical evaluation and lab parameters related to inflammation compared to delayed second-line treatment. Additionally, first-line treatment resulted in less treatment failure and fewer extraintestinal manifestations during TNF-α blockade.

**Conclusion:**

First-line treatment with anti-TNF-α drugs is effective and safe. An earlier start significantly reduces the risk of treatment failure and is associated with fewer extraintestinal manifestations during longitudinal follow-up.

## Introduction

Inflammatory bowel disease is a life-changing diagnosis for many children, adolescents, and their parents. Various treatments have been established to induce and maintain remission with varying efficacy, adherence, and side effects. For two decades, first-line treatment used to induce remission in patients with luminal Crohn’s disease (CD) has been exclusive enteral nutrition (EEN). As a second-line treatment, the biologics infliximab and adalimumab have been recommended in guidelines for inflammatory bowel disease (IBD) in children and adolescents for more than 15 years.

Generally, epidemiological evidence to treat patients with Crohn’s disease presenting high disease activity with infliximab and adalimumab is significant (1). Infliximab (IFX) is associated with mucosal healing after 3 months of treatment (2) and an improvement in clinical disease scores. C-reactive protein levels and blood cell counts are normalized in children and adolescents with moderate to severe CD (3). However, compared to EEN, biological treatment is similarly effective in the induction of mucosal remission in patients with CD and improves quality of life (4, 5). In a review of clinical studies, the long-term therapeutic benefit of IFX measured by continuous therapy in pediatric patients with CD was described (6). Accordingly, significant mucosal healing was observed in patients with CD who were treated with adalimumab (7). Approximately 60% respond well to anti-TNF-α treatment and 40% achieve sustained remission, according to a review of randomized controlled trials and other smaller trials (8). Scarallo and colleagues identified that biologics promote mucosal and histological healing in approximately 40% of pediatric patients with Crohn’s disease and ulcerative colitis (UC) (9). Remission at the end of induction was a predictor of long-term mucosal healing (9). In pediatric patients with UC, treatment with IFX is independently associated with a reduced need for colectomy surgery compared to other treatment options. These treatments had no effect on the number of hospital admissions in general (10).

In Germany, other biologics are available for off-label use. In a retrospective multicenter study, approximately 54% of pediatric patients with IBD who were treated with vedolizumab achieved remission after 14 weeks, even after treatment failure with anti-TNF-α drugs (11). In another small multicenter sample, ustekinumab led to endoscopic improvement in children with UC who were refractory to therapy with infliximab and vedolizumab (12). Similarly, golimumab was an effective treatment for IBD in children and adolescents because it induced clinical remission, as measured by clinical scores (13). Ninety percent of patients naïve to biological agents who were treated remained steroid-free compared to 50% of the patients who failed to respond to other biological therapies (13).

Recent guidelines recommend the prescription of initial biological agents such as infliximab as first-line therapy and adalimumab as an alternative treatment option for pediatric patients with extensive Crohn’s disease. Ustekinumab and vedolizumab are subsequently recommended for consideration when other treatments, including anti-TNF-α treatment, fail (14, 15).

A large cohort study shows that independent of the IBD subtype, the administration of biologics less than 120 days after diagnosis is associated with treatment with fewer glucocorticoids (16). In a retrospective Canadian study, earlier initiation of anti-TNF-α treatment was associated with an older age, higher PCDAI/PUCAI and lower serum albumin levels at diagnosis in patients with CD and UC (17). Evidence implicates a benefit of **early treatment with biological agents** for pediatric patients with Crohn’s disease (18, 19). Disease progression and the development of disease complications, such as stricturing or penetrating behavior, were significantly prevented due to early and effective intervention (19). In a large cohort study of children, early treatment with anti-TNF-α drugs within 3 months after diagnosis seemed superior to treatments with or without immunotherapy regarding the achievement of remission within 1 year (18). In a multicenter randomized prospective trial that investigated moderate to severe Crohn’s disease, a significantly greater proportion of patients treated with a first-line anti-TNF-α therapy achieved clinical and endoscopic remission (20). Additionally, this treatment required significantly less dose escalation and still achieved mucosal healing (20). In a cohort study in Australia, early treatment with infliximab (within 12 months) was shown to be equally cost-efficient but with better clinical outcomes than later treatment with infliximab (21).

### In daily clinical routine, primary and secondary treatment failure is observed and represents a challenge

In a small retrospective study, Cozijnsen and colleagues reported remission in 2/3 of patients treated with adalimumab after failing to respond to infliximab treatment (22). A large retrospective registry study of adult patients with *ulcerative colitis* showed that 50% of patients with UC have a suboptimal response to anti-TNF-α agents, resulting in dose escalation and discontinuation (23).

The current available literature suggests that treatment with anti-TNF-α agents is associated with a very low risk of developing malignancies, which almost exclusively occur when these agents are used in combination with azathioprine (24). In a small sample, adverse events related to TNF-α therapy occurred in 32% of pediatric patients with Crohn’s disease (25). Infusion reactions were reported (5%), and psoriatic rash was reported in eleven percent of the patients. Minor infections also occurred in 15.4% (25). More serious adverse events have been reported in single case studies. In two patients with UC, Pott’s puffy tumor (26) or persistent and productive cough (27) was described in response to treatment with vedolizumab.

The aim of this study is to investigate the use of biological agents in a large sample of children and adolescents with IBD in Germany and to describe (1) the clinical characteristics of these children, (2) clarify the dose and medication of the biologics, and (3) the specific prescription behavior for first-line and second-line prescriptions.

## Methods

Patients with a diagnosis of Crohn’s disease, UC or IBDu (unclassified type of IBD) aged up to 18 years who were treated in specialized IBD centers in Germany or Austria and registered in the CEDATA-GPGE registry were retrospectively analyzed using the prospectively collected registry data. Fifty-two hospitals, clinics, and practices in Germany and Austria submitted clinical and paraclinical data, and appropriate written consent and ethical approval were obtained [University Hospital of Leipzig, University Hospital of Giessen (AZ 74/21) and the Medical Faculty of the Friedrich-Alexander University Erlangen (#301_19Bc)]. For a detailed description of the data structure and history of the CEDATA-GPGE registry, see the study by Buderus et al. (28).

### Inclusion criteria

In the first step, patients fulfilling the inclusion criteria according to the CEDATA working group were considered. All patients registered before 2016 with a period longer than 2 weeks between the first registration and the completed first documentation, as well as patients with more than 1 year between the date of diagnosis and entry into the registry were excluded.

**FIGURE 1:**
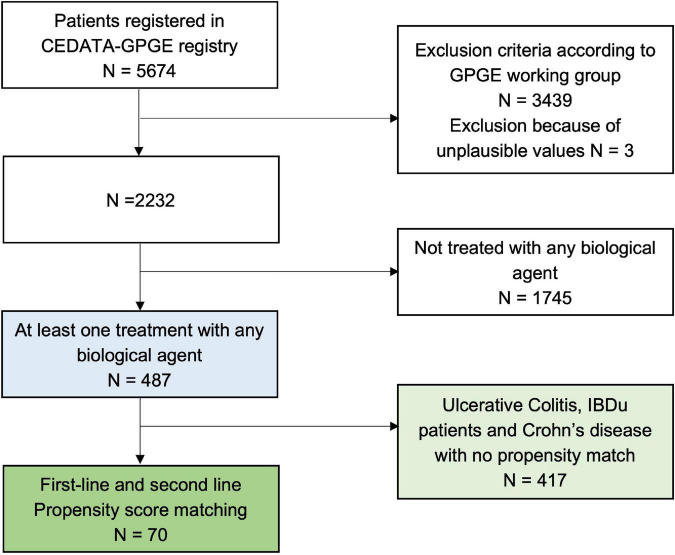
Flow chart of patients registered in the CEDATA registry who were included in this study.

In a second step, patients from the CEDATA registry who met the abovementioned criteria and received a biological agent as treatment (infliximab, adalimumab, vedolizumab, ustekinumab, tocilizumab, or golimumab) were selected. See Figure 1 for a detailed overview of the step by step inclusion process.

### Measures and statistical parameters

Standard clinical information was collected by clinicians at the date of presentation.

Data were collected using two different reporting forms, one for the initial presentation and the second for all follow-up examinations [for detailed information, see Buderus et al. (28)]. Since 2016, data have been submitted *via* online forms.

The initial form included demographic information, including family history, interval from initial symptoms to diagnosis, and symptoms (intra- and extraintestinal manifestations). Information was obtained in a parental report. For a detailed description of the assessed aspects in CEDATA, see the study by Timmer et al. (29). Follow-up examination forms were completed when the patients presented to a participating institution at least twice a year to document illness activity and gather clinical and paraclinical information. The follow-up form also includes medication (type, date of initiation, dose, possible side effects, and end of medication along with the reason), disease-associated complications (e.g., surgery) and laboratory parameters [calprotectin, erythrocyte sedimentation rate (ESR), C-reactive protein (CrP), leukocyte count, hemoglobin, thrombocyte count, lipase, alanine transaminase (ALT), gamma-glutamyltransferase (GGT), and albumin levels]. Additionally, the pediatric Crohn’s disease activity index (PCDAI), short PCDAI (30) and UC activity index (PUCAI) were evaluated based on the assessed data (31). Additionally, the physician’s Global Assessment (PGA) score was recorded. If Paris classification criteria were missing, they were assessed through endoscopy findings (entered separately into the registry) (32).

Patients were considered to receive a first-line biological agent when the diagnosis and prescription of anti-TNF-α medication occurred in the same month. Second-line treatment was considered if conventional remission-inducing therapy, such as ENT or glucocorticoids, failed or physicians decided to use biologics because of flares under conventional remission-preserving therapy with azathioprine, mesalazine or methotrexate.

The CEDATA registry incompletely assesses predictors of poor outcomes for Crohn’s disease (POPO) (14). However, the criteria following criteria are included: (2) no remission after adequate induction therapy, (3) at least L3 at the first diagnosis according to the Paris classification, (4) significant growth retardation (*P* < −2.5 SDS), (6) fistulizing or stricturing disease activity, and (7) perianal disease at diagnosis. *Treatment failure* was defined as one the occurrence of the following events after 1 month of treatment with anti-TNF-α agents: (1) switch or stop of biologics, (2) the need for surgery, (3) initiation of a simultaneous or subsequent systemic application of steroids, (4) clinical scores greater than the cutoff for severe disease activity (PUCAI ≥ 65; PCDAI short ≥ 30), or (5) physicians’ global assessment of high disease activity.

### Data analysis and statistics

Data were analyzed using IBM SPSS statistics 27, Armonk, NY (33). Descriptive analyses examined the usage of biological agents and patient characteristics, such as age, sex, type of IBD and clinical parameters. For normally distributed metric data, the means and standard deviations (M ± SD) are reported separately for patients with Crohn’s disease, UC and unclassified IBD. Medians and ranges are reported for non-normally distributed data or subgroups with small sample sizes (*N* < 7).

Differences in prescriptions were analyzed by comparing patients who received a biological agent at least once with those never receiving a biologic, patients with the different IBD subtypes and patients receiving biological agents as first-line treatment or as second-line treatment. *T*-tests were used to compare the groups when diagnosed (t_0_), and ANOVA was performed to compare subtypes of IBD (t_0_). Propensity score matching was performed to compare first-line and second-line prescriptions of biological agents in patients with Crohn’s disease patients. Due to the small numbers of patients with UC and IBDu receiving biologics as first-line medication, these groups were excluded from the analysis. Propensity scores were estimated based on age, sex, height, weight, diagnosis, inflammation-related parameters (CrP level), Paris classification at t_1_, and the time from the first prescription to the next appointment (t_1_ to t_2_ in months). Multivariate repeated-measures ANOVA compared the clinical course after the prescription of biological agents, analyzing two measurement points, t_1_ when starting treatment with biological agents and t_2_ as the next recorded measurement after prescription.

Prescriptions and changes in biological agents are displayed as a Markov chain model (Figure 3). Every measurement for a patient who received a biological agent was considered, and the probability of represcription or change was calculated as follows:, e.g., p(IFX to adalimumab) = (N_change from IFX to adalimumab_/N_all measurements_) *100%.

**FIGURE 2:**
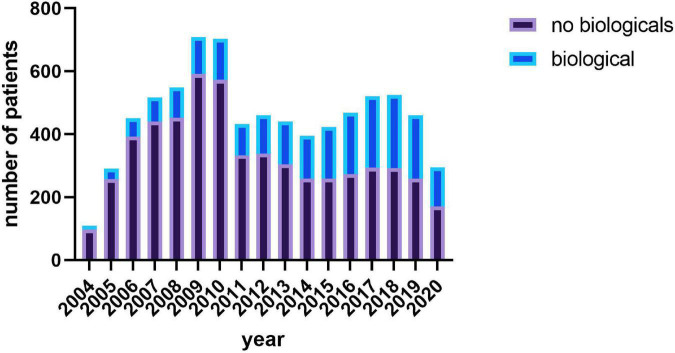
Number of patients receiving a biological agent year.

**FIGURE 3:**
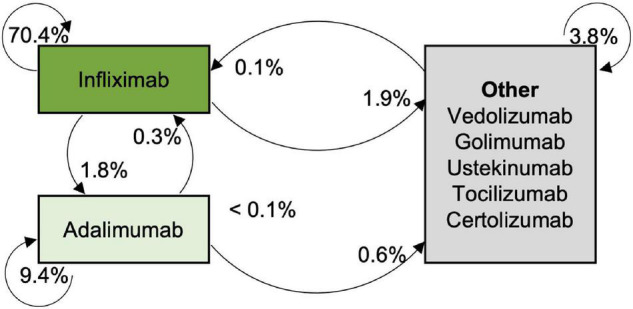
Probabilities for maintaining or changing the biological agent.

Extraintestinal manifestations and side effects were clustered in groups. The association of IFX dosage and side effects was evaluated using the Chi^2^ test. Predictors of extraintestinal manifestations were investigated from the time after the patient first received a biological agent. Cox regression analyses were performed to evaluate risk factors for treatment failure and identify predictors of extraintestinal manifestations, such as age, sex, time since diagnosis in months, first-line prescription, and a high starting dose of infliximab (> 10 mg/kg).

Dependent variables were assessed for a normal distribution visually and using Shapiro–Wilk tests. Extreme values were identified and removed from the analysis. Metric testing was performed thereafter. Comparisons of subgroups with a size smaller than seven were abandoned. The alpha level was set to 5%. Effect sizes are reported according to the tests and interpreted according to Cohen (partial η^2^: 0.01–0.06 small effect, < 0.14 medium effect, > 0.14 large effect; R^2^: 0.02–0.13 small effect, > 0.26 medium effect, > 0.26 large effect) (34).

## Results

### Baseline characteristics

Four hundred eighty-seven patients were treated with biological agents at least once (see Figure 1 highlighted in blue). Those patients were approximately 12 years old (11.9 ± 3.3 years; range 1.4–17.4 years), had a mean height of 150 cm (SDS: −0.1 ± 1.4; range −4.1–8.6) and a mean weight of 39 kg (SDS: −0.8 ± 1.7; range 3.4–14.4). A total of 59.1% were boys (*N* = 288). The diagnoses when entering the registry were *Crohn’s disease* (*N* = 348) in 71.5%, *ulcerative colitis* (*N* = 106) in 21.8% and *unclassified IBD* (*N* = 33) in 6.8% of the patients.

Patients entered the registry between June 2004 and October 2020. On average, 12 reports per patient were submitted (12.3 ± 11; range 1–94) during the period of 30 months (30.1 ± 27.1 months; range 0–149 months). Supplementary Table 1 presents the initial disease activity at diagnosis, and Supplementary Table 2 presents baseline differences between patients receiving a biologic and patients who never received a biologic. Patients receiving a biological agent during their time in the registry were significantly younger and had a higher disease activity, even when entering the registry. The percentage of patients receiving biological agents increased over the years (see Figure 2).

### Disease activity at biological drug prescription

*Crohn’s disease* activity was moderate on average (short PCDAI 29.3 ± 17.9). A total of 20.4% of patients were in remission (short PCDAI < 15), 27.3% had moderate disease activity (15–30) and 52.3% had severe disease activity (≥ 30). Seventy-seven percent fulfilled the criteria for predictors of a poor outcome (*N* = 268, POPO-positive). Most patients were classified as L3L4a according to the Paris criteria (33%, for details, see Table 1). A total of 1.5% had growth retardation. Most had non-penetrating and non-stricturing disease (B1 78.7%), followed by penetrating disease with perianal involvement (B3p 14.4%). Notably, 77.3% were diagnosed with Crohn’s disease between 10 and 17 years of age (A1b), and 21.3% were diagnosed before the age of 10 years (A1a).

**TABLE 1:** Clinical parameters at first prescription.

	Crohn’s disease *N* = 348		Ulcerative colitis *N* = 106		IBDu *N* = 33
PARIS	L1	6.5%	E1	2.2%	
	L2	12.9%	E2	12.9%	
	L3	19.7%	E3	10.8%	
	L4a + L1-L3	51.4%	E4	74.2%	
	L4b + L3	0.9%			
	L4ab + L1-L3	7.2%			
**Physicians’ global assessment**					
Remission	6.5%		13.6%		9.7%
Light activity	26.3%		19.3%		25.8%
Moderate activity	47.7%		45.5%		41.9%
Severe activity	19.5%		21.6%		22.6%

L = Location (L1 = terminal ileum, L2 = colonic, L3 = ileocolonic, L4a = Upper disease proximal to ligament of Treitz, L4b = Upper disease distal to ligament of Treitz); E = Extend (E1 = proctitis, E2 = left-sided colitis, E3 = extensive colitis, E4 = pancolitis).

Disease activity measured with the pediatric *ulcerative colitis* activity index was mild on average (PUCAI 35.5 ± 24.1). A total of 19.8% of patients were in remission, 31.1% had mild disease activity, 37.8% had moderate disease activity, and 11.3% had high disease activity. Most patients were diagnosed with pancolitis (see Table 1). A total of 34.9% had a PUCAI score > 65 points at least once (Paris S1 classification).

### Application of biological agents

On average, patients were 13 years old (13.7 ± 2.8 years) when first receiving a biological agent approximately 19 months after initial diagnosis (19.1 ± 21.2 months; range 0–129 months). Physicians most commonly prescribed infliximab as the first choice among biological agents (88.5%), followed by adalimumab (10.9%), vedolizumab (0.4%), and golimumab (0.2%, *N* = 1). 87.4% of the patients with Crohn’s received infliximab first, 12.3% adalimumab and one patient Golimumab. In UC infliximab was given to 92.9% firstly, 6.1% received adalimumab and one patient vedolizumab. For IBDu patients 87.5% infliximab was administered first, 8.2% had adalimumab and one patient vedolizumab again. Between the IBD-subgroups the choice of the first biological agent was not significantly different (*p* > 0.05). The initial dose of infliximab prescribed to patients with *Crohn’s disease* was 5.8 mg/kg body weight (5.8 ± 1.6, range 1.6–13 mg/kg; 90.1% dosage < 7.5 mg/kg, 9.3% dosage of 7.5–12.5 mg/kg, 0.6% above 12.5 mg/kg), while 1.1 mg/kg adalimumab was administered to patients as the initial treatment (1.1 ± 1, range 0.1–6 mg/kg). Combination with immunomodulators was common and is recommended for infliximab (15). Immunomodulators were additionally applied in 79% of the cases receiving infliximab [58.9% thiopurine, 11% methotrexate (MTX), 5-Asa 36.4%] and in 68.9% of patients receiving adalimumab (46.7% thiopurine, 26.7% 5-Asa, 18.8% methotrexate) After the induction phase, the infliximab dosage was 6.3 mg/kg body weight (± 1.7, range 1.1–14.9), and 1.1 mg/kg (± 1.1, range 0.1–7.3 mg/kg) for adalimumab, respectively.

Patients with *ulcerative colitis* received 6 mg/kg body weight infliximab (6.2 ± 1.9, range 3.7–12.6 mg/kg, 82.8% dosage < 7.5 mg/kg, 15.6% dosage 7.5–12.5 mg/kg, 1.6% above 12.5 mg/kg), while 1.3 mg/kg adalimumab (1 ± 2, range 0–15 mg/kg) was administered in the induction phase which started 20 months (20 ± 22.2) after initial diagnosis. 90.1% received an immunomodulator additionally to infliximab (56% thiopurine, 73.6% sulfasalazine, MTX 5.5%) and 83.3% additionally to adalimumab (33.3% thiopurine, 66.7% 5-Asa, 33.3% MTX). Mean dosage after induction was 7.1 mg/kg body weight (± 2, range 1–13.4 mg/kg) for infliximab and 1.3 mg/kg in adalimumab (±2, range 0.6–15).

Patients with *unclassified IBD* mostly received infliximab at a dosage of 6.7 mg/kg body weight (6.7 ± 1.9, range 4.6–10.6 mg/kg, 72.2% dosage < 7.5 mg/kg, 27.8% dosage > 7.5 mg/kg). 21.3 months (±21.3) after diagnosis biological agents were applied firstly and infliximab was combined with methotrexate (16.7%), thiopurines (41.7%), and sulfasalazine (45.8%). After induction patients with IBDu received infliximab in a dosage of 7 mg/kg body weight (7 ± 2.2, range 3.4–12.7) and 0.9 mg/kg body weight of adalimumab (0.9 ± 0.3, range 0.5–1.3).

A total of 11.4% of patients were treated immediately after the initial diagnosis (first-line, 0 months after diagnosis; CD: *n* = 47; UC: *n* = 8; IBDu: *n* = 1). In the registry, biological agents were taken for approximately 18 months (±17.9; range 0–119 months).

Notably, 24.9% of all infliximab-treated patients received a dosage ≤ 5 mg/kg, and 4.7% received a dose > 10 mg/kg. Adalimumab was administered to most patients at a dosage of 40 mg (independent of weight). In general, the application of biological agents was increasing over the last 10 years reaching a proportion of up to one third of all IBD patients reported to the registry (Figure 2).

### Choice of biologics

Most of the patients received infliximab at least once (*n* = 442). Adalimumab was administered to 115 patients at least once, whereas vedolizumab (*n* = 21), ustekinumab (*n* = 10), golimumab (*n* = 4), tocilizumab (*n* = 2), and certolizumab (*n* = 2) were administered less frequently. 60.7% of patients received IFX for at least 1 year and 20.4% for 2 years and the remaining patient cohort for even longer. Adalimumab was given to 61.4% for at least 1 year and to 25.2% for at least 2 years. Ustekinumab was given up to 2 years, golimumab and tocilizumab even for 4 years, respectively.

Some patients received more than one biological agent during the documented treatment. Seventy-seven patients changed the medical treatment with the application of another biological drug once (receiving two biological agents consecutively), 11 of them changed twice, and one each changed four times and five times (see Figure 3 for probabilities and changing patterns of biological agents). Specific reasons to switch the biological agent were provided by the physicians in 18.6% of the cases with more than one reason possible. Of these reasons, side-effects were most common stated (8.9%), followed by treatment failure (7.1%). Anti-drug antibodies were documented in 3.5% of the cases and allergic reactions in 1.8% (for further details see Supplementary Table 3). 24.3 months (24.3 ± 20.7, range 0–102 months) after the first application of a biological agent, the next one was applied.

### Off-label application of biological drugs

Thirty-one patients (6.37%) received at least one off-label biological agent.

***Vedolizumab*** was administered to 21 patients in the registry. These patients received vedolizumab once to 15 times. These patients were 14 years old (14.2 ± 3.3 years), 160 cm tall (Q1 = 147.3; Q2 = 160.5; Q3 = 169.5) and weighed 48 kg (Q1 = 37.8; Q2 = 47.6; Q3 = 57.8). Vedolizumab was prescribed approximately 4 years after diagnosis (52.9 ± 22.9 months) and 2.5 years after first receiving a TNF-α blocker (32.2 ± 17 months, range 3–78 months). **None of these patients were anti-TNF-**α **therapy-naïve.** The patients received vedolizumab at 5.6 ± 4.8 measurement points (range 1–15). Seven patients had *Crohn’s disease*, and 14 patients had *ulcerative colitis*. The diagnosis of one patient in each of these groups changed to *IBDu* during treatment. Most patients received a dose of 5.4 mg/kg (Md, range 1–13.9).

Ten patients received ***ustekinumab*** 1–8 times. All patients had *Crohn’s disease.* Patients were 15 years old (Md = 15.3), 164.5 cm tall (Q1 = 161.1; Q2 = 164.5; Q3 172.3) and weighed 48.8 kg (Q1 = 45.03; Q2 = 48.8; Q3 = 66.5). Ustekinumab was prescribed 95.5 months after diagnosis and 42.5 months after receiving a biological treatment. During application, the typical dosage was 90 mg.

***Golimumab*** was prescribed to four patients who applied it one to eight times, and 75% of those patients had *Crohn’s disease*. Golimumab was administered 33 months after diagnosis and 16 months after first receiving a biological agent. Most patients took 50 mg (Md, range 50–100 mg).

Tocilizumab and certolizumab were administered to one patient each with *Crohn’s disease*. ***Tocilizumab*** was prescribed 85 months after diagnosis and 78 months after first receiving a biological agent. ***Certolizumab*** was administered to the patient 95 months after diagnosis and 52 months after first receiving a biological agent. Both medications were prescribed 3 times in the registry.

### First-line vs. second-line biological therapy

Patients with *Crohn’s disea*se receiving a biological agent as first-line therapy showed higher disease activity according to the short PCDAI scores (1st line 33.7 ± 15.9, 2nd line 16.7 ± 17.4; *t* = −6.359, df = 363, *p* < 0.001, *d* = 0.992) and doctors’ global assessment (*t* = −7.061; df = 333, *p* < 0.001, *d* = 1.103), with large effect sizes. Significantly more patients fulfilled the criteria for POPO positivity (95.6%) when receiving a biological agent as first-line medication (Fisher’s exact test, Chi^2^ = 9.872, *p* < 0.001). Lab parameters differed significantly between patients receiving first- and second-line treatment due to the number of thrombocytes, leukocytes, hemoglobin, and albumin (see Table 2). Inflammation was more intense in first-line-treated patients, as indicated by higher CrP, ESR, and calprotectin levels (see Table 2).

**TABLE 2:** Significant differences in lab parameters between patients with *Crohn’s disease* receiving 1st-line and 2nd-line treatment.

Crohn’s disease	1st line (*N* = 47)	2nd line (*N* = 318)		
Thrombocytes	453.72 ± 111.42	359.97 ± 110.99	*T* = −5.186, df = 307, ***p* < 0.001**	*d* = 0.810
Leukocytes	9.28 ± 3.44	7.49 ± 9.73	T = −2.410, df = 319, ***p* = 0.016**	*d* = 0.377
Hemoglobin	6.91 ± 1.06	7.58 ± 0.98	*T* = 3.836, df = 326, ***p* < 0.001**	*d* = 0.599
Albumin	37.86 ± 5.92	42.54 ± 5.49	*T* = 4.552, df = 239, ***p* < 0.001**	*d* = 0.711
ESR	34.88 ± 20.39	19.66 ± 16.13	*T* = −4.287, df = 256, ***p* < 0.001**	*d* = 0.670
CrP	42.32 ± 39.15	17.75 ± 36.91	*T* = −4.100, df = 315, ***p* < 0.001**	*d* = 0.641
Calprotectin	679.21 ± 370.28	378.43 ± 377.77	*T* = −3.473, df = 239, ***p* = 0.001**	*d* = 0.543

The bold values are represent p-values < 0.05.

Eight patients with *ulcerative colitis* received a biological agent as a first-line treatment and 90 received it as a second-line treatment. First-line-treated patients had significantly higher disease activity as assessed using the PUCAI score (*t* = −2.655; df = 96, *p* = 0.009, *d* = 0.415) and as determined by doctors (*T* = −2.713, df = 82, *p* = 0.008, *d* = 0.424). ESR was significantly faster in patients receiving first-line treatment (*t* = −3.074; df = 69, *p* = 0.003, *d* = 1.582), and other lab parameters did not differ significantly. Comparisons between first-line and second-line prescriptions were not performed for patients with *IBDu* due to the small sample of patients receiving first-line prescriptions.

### Propensity score matching evaluation of the treatment effect of biologicals when used as a first- or second-line treatment in patients with Crohn’s disease

Using propensity score matching according to the requirements described previously (18), two groups of 35 patients each with *Crohn’s disease* were identified for a comparison of the clinical course.

After propensity score matching, patients in both groups did not exhibit significant differences in the calprotectin level, liver enzyme levels, lipase level, CrP level, hemoglobin level, dose of biologicals or time since the last visit. Nevertheless, patients receiving anti-TNF-α medication as a first-line therapy displayed significantly lower albumin levels, higher inflammation according to leukocyte counts and ESR, higher thrombocyte counts and lower hematocrit (all *p* < 0.05).

**FIGURE 4:**
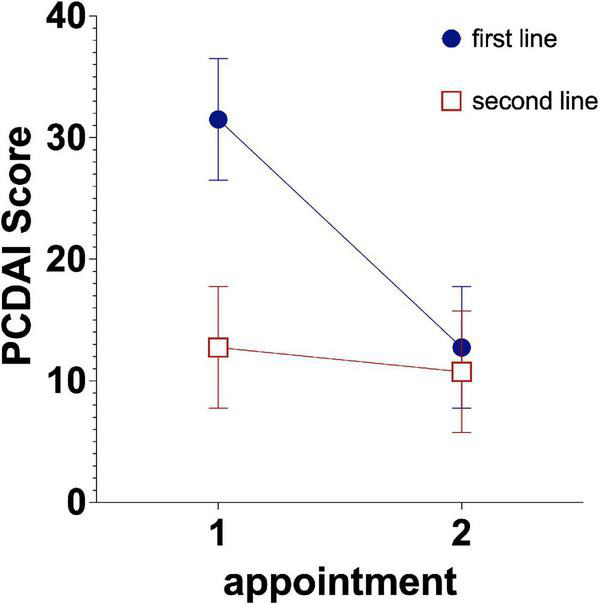
Development of disease activity in patients receiving first- and second-line treatment. Estimated mean PCDAI short scores are shown with the 95% CIs.

Patients with Crohn’s disease displayed higher disease activity when receiving a biological agent as a first-line treatment. At the next appointment (mean of 3 ± 2.7 months after prescription, range 1–11 months, *T* = −1.130, df = 68, *p* = 0.262), the disease activity assessed using PCDAI was similar to that of patients receiving a biological agent as a second-line treatment (see Figure 4). An analysis of lab parameters (albumin level and thrombocytes) produced similar results (see Table 3). Liver enzyme (GGT and ALT) levels improved in both groups over time [*F*(2,54) = 3.257; *p* = 0.045; partial η^2^ = 0.110]. Significant main effects or interaction effects of treatment were not observed on lipase levels, inflammation-related parameters (calprotectin level, CrP level, ESR, leukocyte counts), hematocrit and hemoglobin levels (*p* > 0.05). The infliximab dosage was equal in both groups over time (*p* > 0.05).

**TABLE 3:** Comparison and clinical course of patients with Crohn’s disease receiving a biological agent as a first- and second-line treatment.

Crohn’s disease	Time effect	Group effect	Interaction effect
Clinical assessment PCDAI + physicians’ assessment	*F*(2, 65) = 1.664, *p* = 0.197	*F*(2, 65) = 8.762, ***p* < 0.001** partial η^2^ = 0.212	*F*(2, 65) = 10.250, ***p* < 0.001** partial η^2^ = 0.240
Inflammation leukocytes/CrP	*F*(2, 56) = 0.529, *p* = 0.593	*F*(2, 56) = 2.646, *p* = 0.080	*F*(2, 56) = 1.346 *p* = 0.269
Thrombocytes	*F*(1, 63) = 0.333, *p* = 0.566	*F*(1, 63) = 12.617, ***p* < 0.001** partial η^2^ = 0.167	*F*(1, 57) = 5.287, ***p* = 0.025** partial η^2^ = 0.077
Albumin	*F*(1, 37) = 0.027, *p* = 0.871	*F*(1, 37) = 10.011, ***p* = 0.003** partial η^2^ = 0.213	*F*(1, 35) = 6.794, ***p* = 0.013** partial η^2^ = 0.155

The bold values are represent p-values < 0.05.

### Corticosteroid free remission

After 1 year, 82.7% of the patients that received anti-TNF-therapy achieved corticosteroid free remission, after 2 years 87% of the patients remained in the registry and after 5 years 88.9%. In total 79.9% of patients were corticosteroid free during or after therapy with biologicals at the end of their documented measurements in the registry. The median time to reach corticosteroid free remission was 2 months. 75.9% of Crohn’s patients received immunomodulators as co-medication (54.3% thiopurine, 34.4% 5-Asa, 11.9% MTX), 80% of UC patients (43% thiopurine, 67% 5-Asa, 8% MTX) and 80% of IBDu patients (50% thiopurines, 40% 5-Asa, 20% MTX) when exhibiting corticosteroid free remission. First-line receiving MC patients received corticosteroid free remission in 95%, CU patients in 62.5%, and none of the IBDu patients when treated first-line.

### Treatment failure

**FIGURE 5:**
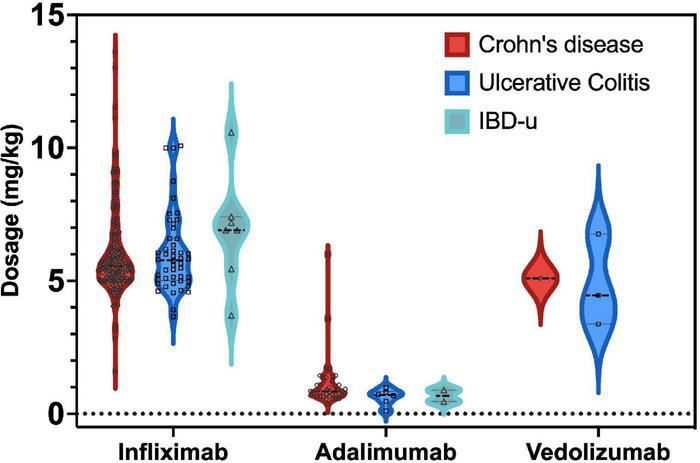
Mean dosage (mg/kg) of biological agents administered at the first occurrence of remission. No mean dosages were available for patients with CD and IBDu receiving vedolizumab, ustekinumab and golimumab due the small number of patients treated at first remission (*n* < 4 per group).

In a Cox regression analysis, different modes of prescription were evaluated to assess treatment failure (Table 4). An earlier start of medication with biologicals and first-line use significantly reduced the probability of treatment failure, whereas using an initial dose > 10 mg/kg was associated with treatment failure. This result was independent of whether the initial dose > 10 mg/kg was used as first- or second-line therapy or as intensified infliximab therapy from 5 to 10 mg/kg in a step-up fashion. Higher albumin levels, younger age and female sex were protective against treatment failure. The dosage at the first occurrence of remission is shown in Figure 5.

**TABLE 4:** Cox regression analysis of treatment failure depending on the parameters of the first prescription.

	Log	SE	Hazard ratio	95% CI		Significance
Age	−0.118	0.011	0.888	0.870	0.907	***p* < 0.001**
Sex	0.161	0.068	1.175	1.029	1.341	***p* = 0.017**
Time since diagnosis	0.009	0.002	1.009	1.006	1.012	***p* < 0.001**
First-line prescription	−0.724	0.127	0.485	0.378	0.622	***p* < 0.001**
High starting dose (IFX)	0.520	0.087	1.682	1.419	1.995	***p* < 0.001**
albumin	−0.085	0.007	0.919	0.906	0.932	***p* < 0.001**

Chi^2^ (4) = 186.296, p < 0.001. The bold values are represent p-values < 0.05.

Calprotectin levels were significantly higher at the time of treatment failure compared to other measurements during the treatment with biologicals [mean = 399.7 ± 389.5 vs. 299.2 ± 362.6 μg/g feces, *F*(3, 467) = 21.745, *p* < 0.001, partial η^2^ = 0.011]. *When considering the IBD subtypes no specific association with calprotectin levels or treatment failure was found.*

### Frequency of extraintestinal manifestations and side effects

During treatment with biologicals, no extraintestinal manifestations were reported for 365 patients. However, 122 patients (25.1%) receiving a biological drug suffered from extraintestinal manifestations of IBD beginning after their first infusion and up to 12 measurement points (Md = 1). After 6 months, this number was further reduced to 86 patients (17.7%) with extraintestinal manifestations. Up to 3 different types of extraintestinal manifestations per patient were reported. Without biological treatment, 130 patients had extraintestinal manifestations of their disease (before treatment: 26.7%). In patients with Crohn’s disease, manifestations in the musculoskeletal system occurred most frequently (*N* = 61, 17.5%), 28 (8%) had cheilitis, liver manifestations were reported in 12 (3.4%), 11 (3.2%) had eye problems (eye, uveitis, iritis, and episcleritis) and two patients (0.6%) experienced skin problems. Twenty-two patients with UC (20.8%) had musculoskeletal manifestations, 4 suffered from liver cirrhosis due to primary sclerosing cholangitis (PSC; 3.8%), two had cheilitis (1.9%), and two patients (1.9%) were reported to have elevated lipase levels without overt pancreatitis. In patients unclassified IBD, liver manifestations were most frequently reported (*N* = 2, 6.1%), and one patient each developed musculoskeletal problems and cheilitis (3%). For a detailed list of extraintestinal manifestations, see Supplementary Table 4).

**FIGURE 6:**
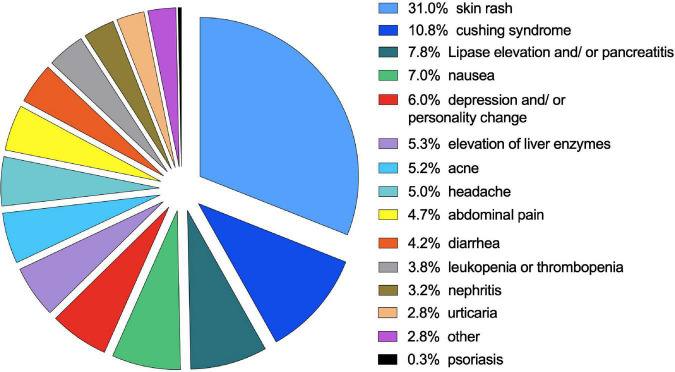
Occurrence of side effects during biological treatment. The percentage of side effects represents all side effects not across all events occurring during treatment with a biological agent.

Side effects were reported in *N* = 221 patients (45.4%) during treatment with anti-TNF-α medication (see Figure 6). Most commonly, skin rash (12.3% of patients), Cushing’s syndrome (8.6%), nausea (6.2% of patients) and elevated levels of liver enzymes (5.4% of patients) were documented. Psoriatic skin rash occurred in 0.3% of patients. For a detailed list of side effects, see Supplementary Table 2. The occurrence of side effects was equal between infliximab and adalimumab (Chi^2^ = 3.266, df = 1, *p* = 0.072). Slightly more side effects occurred when patients were treated with adalimumab (18.3%) than infliximab (15%). Different dosage regimens (< 5 mg/kg vs. 5–10 mg/kg vs. > 10 mg/kg) were not associated with differences in the side-effect occurrence (*p* > 0.05).

In a Cox regression analysis of the occurrence of any extraintestinal manifestation, first-line prescription of biologics significantly prevented extraintestinal manifestations (see Table 5). A younger age when starting with biological agents was also associated with a lower occurrence of extraintestinal manifestations.

**TABLE 5:** Cox regression analysis of the occurrence of extraintestinal manifestations depending on the parameters of the first prescription.

	Log	SE	Hazard ratio	95% CI		Significance
Age	−0.101	0.019	0.904	0.871	0.938	***p* < 0.001**
Sex	−0.010	0.117	0.990	0.787	1.244	*p* = 0.929
Time since diagnosis	−0.007	0.004	0.993	0.986	1.001	*p* = 0.084
First-line prescription	−1.153	0.175	0.316	0.224	0.444	***p* < 0.001**
High starting dose (IFX)	0.122	0.142	1.130	0.855	1.493	*p* = 0.392

Chi^2^ (5) = 62.503, p < 0.001. The bold values are represent p-values < 0.05.

## Discussion

The analysis of a large German/Austrian multicenter registry sample receiving biologicals shows that biological agents have been commonly used to treat IBD in children and adolescents within the last 16 years. Patients with all subtypes of IBD received biological agents mainly when disease activity was high. Most patients received infliximab followed by adalimumab, and approximately 6% of the patients were treated with an off-label biologic at least once. Around 13% of patients with Crohn’s disease had very high disease activity upon diagnosis and received an anti-TNF-α blocker as a first-line treatment. Those patients benefitted significantly, as shown in a propensity score matching analysis of the course of laboratory parameters (albumin levels and thrombocyte counts) and clinical assessment (PCDAI) compared to second-line prescription. The observation that first-line prescription of an anti-TNF-α blocker significantly prevented treatment failure when first receiving a biological agent is consistent with this finding. Extraintestinal manifestations were documented in 17% of patients, most often involving the musculoskeletal system (8.6%). Side effects of anti-TNF-α inhibitor therapy occurred in around 45% of patients, most frequently skin rashes (12.3%).

High disease activity was reported equally in patients with Crohn’s disease, UC and unclassified IBD when first receiving a biological agent. This fact confirms the appropriate physicians’ assessment and indicates that the prescription behavior is related to the disease activity and a correct indication for medical treatment. These observations are consistent with a European survey of ESPGHAN members in which infliximab was most commonly prescribed as a first-line treatment (35).

Changes from infliximab to adalimumab were most common (1.8% of all events). When changing biological treatment, one must consider that research indicates that patients who no longer respond to infliximab benefit more from adalimumab treatment than patients who never responded to infliximab (22). The risk of a suboptimal response was higher in patients were treated with adalimumab and golimumab as the 2nd choice among the anti-TNF-α drugs when compared to infliximab (23).

In addition to malnutrition, hypoalbuminemia indicates a protein-losing enteropathy due to a high level of mucosal inflammation. A subgroup analysis of treatment failure revealed that hypoalbuminemia was associated with anti-TNF-α treatment failure, most likely due to intestinal biological drug loss.

Among the off-label biologicals used in the present study, vedolizumab was used most often in German/Austrian pediatric patients with IBD, but exclusively after failure of treatment with other biological agents. Vedolizumab was slightly more commonly prescribed to patients with UC, whereas other off-label biologics were mostly administered to patients with CD. None of the patients receiving one of these biologics were naïve to TNF-α-blockers. Combination therapy with ustekinumab and vedolizumab successfully led to the closure of fistulas and restoration of bowel continuity in three children with therapy-refractory Crohn’s disease (36). However, a combination of biological agents was not observed in the CEDATA registry.

Although early administration of TNF-α-blockers was shown to be beneficial in pediatric patients with IBD (18, 19), this practice does not appear to be very common yet, as only 11.4% of patients received a biological drug immediately after diagnosis. Those patients showed higher disease activity, and consistent with previous studies, it was associated with higher clinical scores (17) and lower albumin levels, as well as higher inflammatory markers in this study. Even when propensity score matching was performed, patients with high disease activity benefitted the most from first-line treatment in terms of clinical scores, thrombocyte counts and an increase in the albumin level. This result is promising when considering the predictive value of an early treatment response for mucosal and histological healing (9). Other studies suggest that disease severity at diagnosis is a predictor for the need for therapy escalation with TNF-a-blockers and treatment adherence to the medication before the administration of biological agents affects an escalation.

Recently, Jongsma and colleagues published their results of a first randomized head-to-head comparison of top-down infliximab and first-line EEN or corticosteroids (1 mg/kg body weight) in children with moderate to severe Crohn’s disease (20). They demonstrated in concordance with this CEDATA registry that the first-line treatment with IFX was superior to conventional treatment in achieving short-term clinical and endoscopic remission and found a greater likelihood of maintaining clinical remission at week 52 on azathioprine monotherapy. They also concluded that children with moderate-to-severe Crohn’s disease would benefit from the first-line IFX treatment.

Patients with lower therapy adherence require therapy escalation significantly more often (37). In the Cox regression analysis performed in the present study, first-line prescription significantly prevented treatment failure, whereas a high infliximab induction dose was associated with treatment failure. We were unable to clarify whether the observed treatment failure was caused by anti-drug antibodies or protein-losing enteropathy associated with significant intestinal drug loss, thus representing a limitation of our registry data. In particular, higher infliximab trough levels after induction have been shown to predict remission 1 year after the administration of infliximab in studies of pediatric patients with UC and CD (38, 39). In addition, the dosage of the biological drug does not substitute for pharmacokinetics, as the amount of drug applied does not necessarily correspond to the determined drug trough level for several reasons (40).

However, the data registry allowed to determine the specific reasons for the switch of the biological agent as these were provided in 18.6% of the cases from the participating physicians. Of the stated reasons side-effects were most common (8.9%), followed by treatment failure (7.1%). Anti-drug antibodies were documented in 3.5% of the cases and allergic reactions in 1.8%. The first choice of anti-TNF therapy – which was infliximab in 88.5% of the cases – was applied for about 1 year in most patients. This corresponds well with the few current data of a multicenter study from Canada (41). Real world data on achieving of corticosteroid remission in pediatric IBD is as scarce as the information on the duration and efficacy of the anti-TNF therapy aside from the original RCTs (REACH and IMAgINE, respectively) (42, 43). The Canadian registry demonstrated a high durability of infliximab therapy as it was shown for 180 children receiving infliximab for CD, where 86% remained on this therapy for a median of 86 weeks (41). Here, we found that more than 80%, around 87% and almost 90% of the patients that received anti-TNF-therapy achieved corticosteroid free remission after one, after two and 5 years, respectively. In addition, corticoid steroid-free remission was achieved in 3/4 of the cases when combined with an immunomodulator. Accordingly, early treatment with biologicals significantly lowered the risk of developing penetrating but not structuring complications (44).

According to current guidelines, combination therapy with infliximab and immunomodulators in German speaking countries is common (15). Moreover, our data indicate that around 70% of CD patients and around 82% of UC patients receive concomitant immunomodulators also with adalimumab even though this is not specifically recommended This observation is in line with findings of a large pediatric and adult sample in North America (45). The authors concluded a beneficial outcome for both combination therapies concerning treatment failure (45).

In most patients, no extraintestinal manifestations (EIMs) were reported during treatment with biological agents (∼25% of patients receiving a TNF-α blocker had EIMs after initiation). This number is higher than that reported in other studies where the rate of all EIMs in patients with IBD was approximately 14% (46). The explanation for these results may be the longer follow-up of our patient cohort, as some of the patients were investigated almost 5 years after the initial entry in the registry. EIMs were associated with an increased risk of relapse and the need for biological treatment (47, 48). The Cox regression analysis of this large multicenter cohort revealed that the first-line prescription mode significantly lowered the occurrence of EIMs in the longitudinal course. This finding seems very promising because those patients had the highest disease activity before treatment. After treatment induction, the occurrence of EIMs and subsequent systemic inflammation was reduced from approximately 27–25% immediately after the start of treatment, while after 6 months, the number was further significantly reduced to approximately 17%.

In a single-center study examining different biologicals across patients with all subtypes of IBD, adverse events were reported in 47% of patients (47), which is again consistent with the current study in which approximately 45% of patients reported side effects. On the other hand, in a cohort of 89 children with Crohn’s disease, Hradsky et al. reported the occurrence of skin complications during anti-TNF-α treatment in 39% of patients. This number is significantly higher than the value of 12.6% reported in the present study. In particular, psoriatic rash was reported in 11% of patients in the study by Nuti et al. (25). This value was far higher than the two reported cases in this study. No association of dosage and side effects was found here. Off-label use was not associated with more adverse events; as in other studies, vedolizumab and ustekinumab yielded good safety profiles (47, 49).

While this multicenter study reveals several new findings that are of importance for the daily clinical application of biological drugs in pediatric patients with IBD, it also has limitations. (1) Propensity score matching was performed to overcome the missing randomization. (2) Many different clinics across Germany and other German-speaking countries participate in the registry, leading to different approaches and different levels of expertise in the treatment of IBD. (3) Additionally, guidelines changed over time throughout the observation period. Nevertheless, the strength of clinical data involving established routine clinical laboratory chemistry parameters, inflammation scores, physicians’ assessment scores, and a large European sample must be considered a valuable advantage.

In summary, TNF-α blockers are established in German-speaking countries as treatments for patients with IBD, preferentially those with high disease activity. First-line therapy with TNF-α antibodies in pediatric patients with IBD produced a superior clinical response, especially in patients with highly active disease. It significantly reduced the occurrence of EIMs in the longitudinal course. Anti-TNF-a therapy in children is safe, adverse events were mostly mild, and complications such as the development of an adverse psoriasis were observed in approximately 13% of patients. The data of the CEDATA registry provides very strong support to the recommended anti-TNF drug application as primary induction and maintenance therapy in children with a high risk of poor outcomes (15). Moreover, anti-TNF agents should be considered early in the treatment regimen in children with moderate-to-severe disease activity or in those who do not reach clinical and biochemical remission (e.g., fecal calprotectin < 250 μg/g) after induction with EEN or corticosteroids.

In subsequent analyses of data from the CEDATA registry, we expect to report additional details on other aspects of achieving and preserving remission, as well as investigating treatment failure by evaluating drug trough levels and neutralizing anti-drug antibodies.

## Data availability statement

The original contributions presented in this study are included in the article/Supplementary Material, further inquiries can be directed to the corresponding author.

## Ethics statement

The studies involving human participants were reviewed and approved by the University Hospital of Leipzig, University Hospital of Giessen (AZ 74/21) and the Medical Faculty of the Friedrich-Alexander University Erlangen (#301_19Bc). Written informed consent to participate in this study was provided by the participants’ legal guardian/next of kin.

## Author contributions

MeC, AH, AS, KS, and JL conceived the study. MeC, AH, and JL designed the study. The CEDATA-GPGE study group acquired the data. MeC, MaC, JL, and AH analyzed and interpreted the data, drafted the article, and revised it critically for important intellectual content. AH approved the final version of the manuscript. All authors contributed to the article and approved the submitted version.

## Conflict of interest

The authors declare that the research was conducted in the absence of any commercial or financial relationships that could be construed as a potential conflict of interest.

## Publisher’s note

All claims expressed in this article are solely those of the authors and do not necessarily represent those of their affiliated organizations, or those of the publisher, the editors and the reviewers. Any product that may be evaluated in this article, or claim that may be made by its manufacturer, is not guaranteed or endorsed by the publisher.

## The CEDATA working group

Stephan Buderus, Bonn; Philip Bufler, Berlin; Martin Claßen, Bremen; Jan Däbritz, Greifswald; Söhnke Dammann, Stuttgart; Jan de Laffolie, Giessen; Almuthe Christina Hauer, Graz, Austria; Klaus-Michael Keller, Wiesbaden; Andreas Krahl, Darmstadt; Martin Laaß, Dresden; Thomas Lang, Regensburg; Carsten Posovszky, Zürich, Switzerland; Burkhard Rodeck, Osnabrück; Stefan Trenkel, Potsdam.
