# Supplementary material for: Significant advantages for first line treatment with TNF-alpha inhibitors in pediatric patients with inflammatory bowel disease – Data from the multicenter CEDATA-GPGE registry study
Source: Front Pediatr. 2022 Jul 19;10:903677. doi: 10.3389/fped.2022.903677 (PMC9595023; doi:10.3389/fped.2022.903677)
Supplement: Supplementary file 1 [file Table_1.pdf]

Supplemental table 1

	Crohn's disease	Ulcerative colitis	IBDu	ANOVA			
	(N = 348)	(N = 106)	(N = 33)	F	df		Sig.
Age at diagnosis	12.08 ± 2.97	11.75 ± 3.81	10.91 ± 4.10	2.131	2	484	p = 0.120
Height (cm)	149.53 ± 18.29	148.01 ± 23.54	147.64 ± 27.42	0.309	2	474	p = 0.735
Weight (kg)	38.64 ± 14.98	41.17 ± 18.80	42.31 ± 22.47	1.479	2	476	p = 0.229
Doctor's assessment	2.80 ± 0.83	2.75 ± 0.95	2.77 ± 0.92	0.13	2	424	p = 0.878
Hemoglobin (mmol/l)	7.15 ± 0.99	6.61 ± 1.32	6.88 ± 1.51	7.272	2	378	**p = 0.001**
Thrombocytes (Gpt/l)	474.16 ± 145.09	453.10 ± 147.07	386.59 ± 153.83	3.952	2	363	**p = 0.020**
Leukocytes (Gpt/l)	10.08 ± 4.23	10.53 ± 4.44	10.28 ± 4.69	0.353	2	389	p = 0.703
ESR (mm/h)	30.13 ± 17.46	21.38 ± 16.70	29.73 ± 18.83	6.228	2	287	**p = 0.002**
CrP (mg/l)	35.36 ± 45.17	12.24 ± 18.76	36.01 ± 51.54	9.816	2	370	**p < 0.001**
Calprotectin (mg/kg)	698.1 ± 402.38	708.26 ± 338.96	742.73 ± 404.65	0.066	2	122	p = 0.937
Albumin (g/l)	38.03 ± 6.75	41.66 ± 6.88	39.19 ± 7.34	5.786	2	252	**p = 0.003**
ALT (µmol/(s l))	0.31 ± 0.38	0.34 ± 0.40	0.65 ± 0.81	7.976	2	391	**p < 0.001**
GGT (µmol/(s l))	0.28 ± 0.20	0.46 ± 0.84	0.68 ± 0.78	10.102	2	352	**p < 0.001**
Lipase (µmol/(s l))	0.66 ± 0.79	0.92 ± 1.61	0.68 ± 0.78	1.569	2	284	p = 0.210
PCDAI short	29.28 ± 17.88						
PUCAI		35.52 ± 24.14					

Table A.1: Laboratory parameters when entering the registry and differences between diagnoses when entering the registry.
