# Supplementary material for: Significant advantages for first line treatment with TNF-alpha inhibitors in pediatric patients with inflammatory bowel disease – Data from the multicenter CEDATA-GPGE registry study
Source: Front Pediatr. 2022 Jul 19;10:903677. doi: 10.3389/fped.2022.903677 (PMC9595023; doi:10.3389/fped.2022.903677)
Supplement: Supplementary file 2 [file Table_2.pdf]

Supplemental table 2

	nonbiologic group		biologic group		t test		
	N	M ± SD	N	M ± SD	T	df	Sig.
Age at diagnosis	1706	12.34 ± 3.62	487	11.93 ± 3.26	2.405	864.803	**p = 0.016**
Height (cm)	1688	151.87 ± 21.78	479	148.99 ± 20.22	2.596	2165	**p = 0.010**
Weight (kg)	1685	42.76 ± 16.10	481	39.37 ± 16.44	4.05	2164	**p < 0.001**
Doctor's assessment	1593	2.48 ± 0.85	429	2.79 ± 0.86	-6.554	2020	**p < 0.001**
Hemoglobin (mmol/l)	1464	7.36 ± 1.18	383	7.03 ± 1.12	5.015	1845	**p < 0.001**
Thrombocytes (Gpt/l)	1249	413.99 ± 142.28	368	464.04 ± 147.07	-5.885	1615	**p < 0.001**
Leukocytes (Gpt/l)	1478	9.63 ± 4.20	394	10.18 ± 4.29	-2.283	1870	**p = 0.023**
ESR (mm/h)	1116	22.28 ± 16.68	293	28.17 ± 17.65	-5.319	1407	**p < 0.001**
CrP (mg/l)	1372	23.03 ± 42.27	376	30.63 ± 42.50	-3.085	1746	**p = 0.002**
Calprotectin (mg/kg)	399	586.13 ± 418.82	126	704 ± 385.43	-2.806	523	**p = 0.005**
Albumin (g/l)	981	40.73 ± 7.20	258	38.92 ± 6.96	3.614	1237	**p < 0.001**
ALT (µmol/(s l))	1432	0.39 ± 0.53	396	0.34 ± 0.44	1.798	1826	p = 0.072
GGT (µmol/(s l))	1313	0.39 ± 0.57	357	0.35 ± 0.48	1.465	1668	p = 0.143
Lipase (µmol/(s l))	1204	0.96 ± 1.66	289	0.73 ± 1.06	2.877	673.4	**p = 0.004**

Table A.2: Differences between patients receiving a biological agent (included in this study) and patients who never received a biological agent when entering the registry
