# Supplementary material for: Significant advantages for first line treatment with TNF-alpha inhibitors in pediatric patients with inflammatory bowel disease – Data from the multicenter CEDATA-GPGE registry study
Source: Front Pediatr. 2022 Jul 19;10:903677. doi: 10.3389/fped.2022.903677 (PMC9595023; doi:10.3389/fped.2022.903677)
Supplement: Supplementary file 3 [file Table_3.pdf]

Supplemental table 3

Reasons for switch	Switches of biological therapy (total n=113)
Reason as specified by the physician	18.6%, multiple reasons are possible
- Allergic reactions	1.8%
- Side effects	8.9%
- Treatment failure	7.1%
- Anti Drug-Antibodies (ADA)	3.5%
Associated events at the same time that have not been specified by the physician	
- side-effects	31.0%
- complications	13.3%
- surgery	1.8%
- endoscopy	19.5%

Table A.3 Reasons for a switch of a treatment with biologicals
