# Supplementary material for: Significant advantages for first line treatment with TNF-alpha inhibitors in pediatric patients with inflammatory bowel disease – Data from the multicenter CEDATA-GPGE registry study
Source: Front Pediatr. 2022 Jul 19;10:903677. doi: 10.3389/fped.2022.903677 (PMC9595023; doi:10.3389/fped.2022.903677)
Supplement: Supplementary file 4 [file Table_4.pdf]

**Supplemental table 4**

| extraintestinal manifestations | number of occasions (N) | total of 588 (%) |
|---|---|---|
| amyloidosis | 31 | 5.3 |
| cheilitis granulomatosis | 7 | 1.2 |
| stomatitis aphthosa | | |
| pyoderma gangrenosum | | |
| skin | | |
| cirrhosis (liver) | 28 | 4.8 |
| liver, pancreas, bile ducts | | |
| liver, PSC | | |
| erythema nodosum | 25 | 4.3 |
| eye, uveitis, iritis, episcleritis | 30 | 5.1 |
| joint pain/-inflammation | 19 | 3.2 |
| spine | | |
| sacroiliitis | | |
| lactose intolerance | 32 | 5.4 |
| migraine, headache | 65 | 11.1 |
| cystic fibrosis | 42 | 7.1 |
| nephritis | 18 | 3.1 |
| pancreas, pancreatic insufficiency | 6 | 1.0 |
| elevated lipase level | 41 | 7.0 |
| other | 6 | 1.0 |

Table A.4: Incidence of extraintestinal manifestations.
